# Intelligence indexes generalist genes for cognitive abilities^☆^

**DOI:** 10.1016/j.intell.2013.07.011

**Published:** 2013-09

**Authors:** Maciej Trzaskowski, Nicholas G. Shakeshaft, Robert Plomin

**Affiliations:** King's College London, Institute of Psychiatry, MRC Social, Genetic and Developmental Psychiatry Centre, London, England SE5 8AF, United Kingdom

**Keywords:** Genome-wide Complex Trait Analysis (GCTA), Twin analysis, Verbal ability, Nonverbal ability, Pleiotropy

## Abstract

Twin research has supported the concept of intelligence (general cognitive ability, g) by showing that genetic correlations between diverse tests of verbal and nonverbal cognitive abilities are greater than 0.50. That is, most of the genes that affect cognitive abilities are highly pleiotropic in the sense that genes that affect one cognitive ability affect all cognitive abilities. The impact of this finding may have been blunted because it depends on the validity of the twin method. Although the assumptions of the twin method have survived indirect tests, it is now possible to test findings from the twin method directly using DNA alone in samples of unrelated individuals, without the assumptions of the twin method. We applied this DNA method, implemented in a software package called *Genome*-*wide Complex Trait Analysis* (GCTA), to estimate genetic variance and covariance for two verbal tests and two nonverbal tests using 1.7 million DNA markers genotyped on 2500 unrelated children at age 12; 1900 children also had cognitive data and DNA at age 7. Because each of these individuals is one member of a twin pair, we were able to compare GCTA estimates directly to twin study estimates using the same measures in the same sample. At age 12, GCTA confirmed the results of twin research in showing substantial genetic covariance between verbal and nonverbal composites. The GCTA genetic correlation at age 12 was 1.0 (SE = 0.32), not significantly different from the twin study estimate of 0.60 (SE = 0.09). At age 7, the genetic correlations were 0.31 (SE = 0.32) from GCTA and 0.71 (SE = 0.15).from twin analysis. The results from the larger sample and stronger measures at age 12 confirm the twin study results that the genetic architecture of intelligence is driven by pleiotropic effects on diverse cognitive abilities. However, the results at age 7 and the large standard errors of GCTA bivariate genetic correlations suggest the need for further research with larger samples.

## Highlights

• Twin research yields high genetic correlations among diverse cognitive abilities.
• A new method based on DNA alone supports these twin study results.
• These ‘generalist genes’ are the genetic foundation for intelligence.

## 1 Introduction

Because intelligence predicts educational attainment, income, health and longevity better than all other predictors combined, it is a key ingredient in the intellectual capital of knowledge-based societies (Deary, 2012). Intelligence is at the pinnacle of the hierarchical model of cognitive abilities that subsumes group factors and specific tests (Carroll, 1993, 1997), which is why it has been called general cognitive ability (g) (Jensen, 1998; Spearman, 1904). Genetic research, largely based on the twin method that compares resemblance for monozygotic and dizygotic twins, suggests that genes with pervasive effects across cognitive abilities are the genetic foundation for intelligence. In contrast to the average phenotypic correlations of about 0.30 between diverse cognitive abilities (Carroll, 1993), genetic correlations among cognitive abilities are consistently greater than 0.50 in more than a dozen studies in childhood, adolescence, and adulthood, with some evidence for increasing genetic correlations during childhood (Plomin, DeFries, Knopik, & Neiderhiser, 2013).

Genetic correlations indicate the extent to which the same genes affect different abilities; they are literally correlations between genetic effects on traits independent of heritability (Plomin, DeFries et al., 2013). This overlap in genetic effects is generally known as *pleiotropy* but has been dubbed *generalist genes* to highlight this finding in relation to cognitive abilities (Plomin & Kovas, 2005). Because genetic correlations are not 1.0, these same data also provide evidence for genetic specificity. However, given how diverse cognitive processes appear to be – such as verbal, spatial and memory – what is surprising is the extent of genetic overlap between these abilities. Although multivariate genetic research has drilled down beneath traditional tests of cognitive abilities to uncover similar results for elementary cognitive processes and brain structure and function (Deary, Penke, & Johnson, 2010), the present paper focuses on traditional tests of verbal and nonverbal cognitive function.

Because generalist genes would seem to be a major finding about the origins of individual differences in cognitive abilities – suggesting that g indexes general genes for cognitive abilities – it is surprising that this finding has had so little impact in related fields such as neuroscience or experimental cognitive psychology. We suggest that part of the reason for this neglect – in addition to the major reason that these fields generally ignore individual differences (Baddeley, 2012; Giedd & Rapoport, 2010) – is that the generalist gene finding rests largely on the twin design, although adoption research also supports the hypothesis. Even though the assumptions of the twin and adoption methods have been tested and generally pass these tests (Plomin, DeFries et al., 2013), these assumptions make it easier to ignore the results of twin and adoption studies.

The purpose of the present study is to investigate the genetic nature of cognitive abilities using a new method that is based solely on DNA, which avoids the assumptions of the twin and adoption methods. The method, implemented in a software package called Genome-wide Complex Trait Analysis (GCTA), correlates genetic similarity pair by pair with each pair's phenotypic similarity in a large sample of unrelated individuals. Specifically, the method partitions the phenotypic variance into additive genetic and residual components by fitting a genetic ‘relatedness’ matrix to a phenotypic matrix in a mixed linear model (MLM; Yang et al., 2010; Yang, Lee, Goddard & Visscher, 2011). Genetic similarity is assessed overall from hundreds of thousands of single-nucleotide polymorphisms (SNPs) in large samples of unrelated individuals; such data are widely available from genome-wide association (GWA) studies (Plomin, 2012). Crucially, unlike GWA studies, this MLM method does not rely on detecting associations with individual SNPs, but rather it calculates the overall effect of all SNPs as well as DNA variants correlated with the SNPs. Because the method does not have a consistent name, we refer to it as GCTA, which is the name of its software package. Univariate GCTA has found genetic influence for intelligence in adults (Chabris et al., 2012; Davies et al., 2011) and children (Plomin, Haworth, Meaburn, Price, & Davis, 2013), as well as for height (Yang et al., 2010) and weight (Yang, Manolio, et al., 2011), psychiatric and medical disorders (Lee, Wray, Goddard, & Visscher, 2011; Lee, DeCandia, et al., 2012; Lubke et al., 2012), and personality (Benjamin, Ebstein, & Belmaker, 2002; Vinkhuyzen et al., 2012).

Bivariate GCTA has recently been developed to estimate genetic correlations between traits (Lee, Yang, Goddard, Visscher, & Wray, 2012). It was first applied to the longitudinal correlation between intelligence in childhood and old age (Deary, Yang, Davies, Harris, Tenesa, Liewald, et al., 2012) and subsequently to childhood intelligence from age 7 to age 12 (Trzaskowski, Yang, Visscher, & Plomin, in press). We have also applied bivariate GCTA to confirm twin study estimates of high genetic correlations between g and academic performance in reading, mathematics and language (Trzaskowski et al., 2013). The present study uses bivariate GCTA to address the fundamental issue of the genetic nature of intelligence itself. We compare GCTA estimates of genetic variance and covariance to estimates from the twin method using the same sample assessed longitudinally at ages 7 and 12 and the same measures of verbal and nonverbal cognitive abilities. Such direct comparisons between GCTA and twin study estimates go beyond merely testing the methodological validity of the twin method: As explained later, they reveal important information about the genetic architecture of intelligence.

## 2 Method

### 2.1 Participants

The sample was drawn from the Twins Early Development Study (TEDS), which is a multivariate longitudinal twin study that recruited more than 11,000 twin pairs born in England and Wales in 1994, 1995 and 1996 (Haworth, Davis, & Plomin, 2013; Oliver & Plomin, 2007). TEDS has been shown to be the representative of the UK population (Kovas, Haworth, Dale, & Plomin, 2007). The project received approval from the Institute of Psychiatry ethics committee (05/Q0706/228) and parental consent was obtained prior to data collection. Individuals were included if their first language was English and they had no major medical or psychiatric problems. Using data collected at ages 7 and 12, respectively, GCTA was conducted on approximately 1900 and 2500 unrelated individuals (only one member of each twin pair) with DNA and cognitive data. Twin model-fitting analyses were conducted on around 1900 twin pairs at age 7 and around 2350 pairs at age 12. As expected for representative twin studies, the twins included similar numbers of MZ twins, same-sex DZ twins, and opposite-sex DZ twins (see Table 1 for sample size details.)

### 2.2 Genotyping

Although DNA is available for more than 12,000 TEDS participants, funds were available to genotype 3665 individuals (one member only per twin pair) on Affymetrix GeneChip 6.0 SNP genotyping arrays using standard experimental protocols. Nearly 700,000 SNPs were genotyped and more than one million other SNPs were imputed using IMPUTE v.2. software (Howie, Donnelly, & Marchini, 2009). These SNPs had survived stringent quality control criteria: allele frequency information > .975, minor allele frequency > .01, genotype call-rate > .80, Hardy–Weinberg equilibrium > 10^− 10^, and plate effect p-value > 10^− 6^. More detailed information can be obtained from the first author. DNA for 3152 individuals survived quality control; of these 3152 individuals, 1900 had cognitive data at age 7 and 2500 at age 12. To control for ancestral stratification, we performed principal component analyses on a subset of 100,000 quality-controlled SNPs after removing SNPs in linkage disequilibrium (r^2^ > 0.2) (Fellay et al., 2007). Using the Tracy–Widom test (Patterson, Price, & Reich, 2006), we identified 8 axes with p < 0.05, which were used as covariates in GCTA analyses.

### 2.3 Measures

The cognitive tests and testing procedures have been described in detail for age 7 (Petrill, Rempell, Oliver, & Plomin, 2002) and age 12 (Haworth et al., 2007). At age 7, testing was conducted by telephone; at age 12, testing was conducted online. At each age, verbal and nonverbal composite scores were derived from two widely used verbal tests and two widely used non-verbal tests.

At age 7, the two verbal tests were the Similarities subtest and the Vocabulary subtest from the WISC-III-UK, and the two nonverbal tests were the Picture Completion subtest from the WISC-III-UK and the Conceptual Grouping subtest from the McCarthy Scales of Children's Abilities. At age 12, the verbal tests included the Information and Vocabulary subtests from the WISC-III-PI Multiple Choice test, and the two non-verbal reasoning tests were WISC-III-UK Picture Completion and Raven's Standard and Advanced Progressive Matrices. All of these tests are reported to have reliabilities greater than 0.80 (McCarthy, 1972; Raven, Raven, & Court, 2003; Wechsler, 1992). Although we have not assessed the reliability of the verbal and non-verbal composites derived from these scales, reliability is of course bound to be even greater for the composites.

For each cognitive measure at each age, scores were regressed on sex and age and standardized residuals were derived, ranked, and quantile normalized (Lehmann, 1975; van der Waerden, 1975) before creating equal-weighted composites for verbal and nonverbal ability. All procedures were executed using R (www.R-project.org). The phenotypic correlations between the verbal and nonverbal composites are 0.35 at age 7 and 0.45 at age 12. Phenotypic correlations between the four subtests at each age are presented in Supplementary data Table 2b.

### 2.4 Twin analysis

The classical twin design and model-fitting, described in detail elsewhere (Plomin, DeFries et al., 2013; Plomin, Haworth et al., 2013), splits phenotypic variance into additive genetic (A), shared or common environmental (C), and non-shared or unique environmental (E) components. Within MZ twin pairs, both genetic and shared environmental effects are assumed to correlate 1.0, whereas within DZ twin pairs, shared environmental effects correlate 1.0 but additive genetic effects only correlate 0.5. Non-shared environmental influences (E) are assumed to be uncorrelated for members of a twin pair and thus only contribute to differences within pairs. Based on previous research and examination of twin correlations, we fit an ACE model with additive genetic, shared environmental and non-shared environmental effects. As is standard in twin analyses, residuals correcting for age and sex were used because the age of twins is perfectly correlated across pairs, which would otherwise be misrepresented as shared environmental influence. The same applies to the sex of the twins, since MZ twins are always of the same sex. Because previous analyses of these data indicated nonsignificant differences in model-fitting results between males and females (Davis, Haworth, & Plomin, 2009; Kovas et al., 2007), we combined same-sex and opposite DZ twin pairs in order to increase the power of the analyses. Twin analyses limited to same-sex twins yielded highly similar results (available from the first author).

We used standard ACE model-fitting analysis in the OpenMx package for R (Boker et al., 2011). Fitting the ACE model for MZ and DZ twins to the data yields estimates of the model's goodness of fit and estimates the contributions of A, C, and E with confidence intervals. We conducted four separate univariate analyses for verbal and nonverbal composites at ages 7 and 12. We also conducted two bivariate analyses for verbal and nonverbal at age 7 and at age 12. For the bivariate analysis, we fit a bivariate Cholesky decomposition using OpenMx, although we present the results from a correlated factor solution because it provides the most direct comparison to GCTA and is mathematically equivalent to Cholesky decomposition (Loehlin, 1996).

### 2.5 Genome-wide Complex Trait Analysis (GCTA)

The GCTA method was applied using the standard GCTA software package (Yang, Manolio, et al., 2011). Conceptually, the GCTA method can be thought of using a matrix of pairwise genomic similarity to predict a matrix of pairwise phenotypic similarity using a random-effects mixed linear model (Yang, Manolio, et al., 2011). Pairwise genomic similarity is calculated between all pairs of individuals in the sample using all genetic markers genotyped on the SNP array or imputed from these SNPs. GCTA uses this Genetic Relatedness Matrix (GRM) to estimate how much of the variance of the phenotypic matrix can be explained by additive effects of the common SNPs on the SNP array or by unknown causal variants correlated with the SNPs. In order to focus on chance genetic similarity required by the random-effects model, for any pair of individuals whose genetic similarity is equal to or greater than a fourth cousin (pairwise relatedness > 0.025), one individual from the pair is removed.

In univariate analysis, the trait's variance is partitioned using restricted maximum likelihood (REML) into genetic and residual components (Yang, Manolio, et al., 2011; Yang et al., 2010). In contrast, the bivariate method extends the univariate model by relating the pairwise genetic similarity matrix to a phenotypic covariance matrix between traits 1 and 2, allowing for correlated residuals (Lee, Yang, et al., 2012). The eight principal components described earlier were used as covariates in our GCTA analyses; all phenotypes were age- and sex-regressed prior to analysis.

## 3 Results

### 3.1 Genetic and environmental influences on verbal and nonverbal abilities: Twin and GCTA estimates

Table 1 presents genetic and environmental univariate estimates for GCTA and twin analyses. At age 12, twin analyses yield moderate estimates of heritability (0.36 for verbal and 0.42 for nonverbal) and modest estimates of shared environmental influence (0.21 for verbal and 0.16 for nonverbal). GCTA estimates of genetic influence at age 12 are significant and on average half (49%) of the twin study heritabilities, although more for verbal (64%) than for nonverbal (36%). GCTA does not distinguish between shared and nonshared environmental influence and it does not detect non-additive genetic influence. That is, all shared and nonshared environmental influence as well as non-additive genetic variance and any genetic variance not capture by the common SNPs on our DNA array are into a residual non-genetic component of variance, which also includes error of measurement.

As compared to age 12, the twin results at age 7 show less heritability (0.29 for verbal and 0.21 for nonverbal) and greater shared environmental influence (0.34 and 0.28). What is unusual about the results at age 7 is that the GCTA estimates are higher, although not significantly so, than the twin study estimates of heritability, especially for verbal ability. However, we ascribe this result to chance and the large standard error for the GCTA estimates, although there are more interesting possible explanations, as discussed later.

More detail about the twin and GCTA univariate results is available online in Supplementary material, including twin correlations and twin model-fitting as well as twin and GCTA results for the individual cognitive tests rather than just their verbal and nonverbal composites.

Stepping back from the details, these results at both ages 7 and 12 show that GCTA estimates of heritability are significant and substantial, thus providing support for the twin study heritability estimates. These findings allow us to proceed to investigate this paper's central question about the genetic correlation between verbal and nonverbal cognitive abilities.

### 3.2 Genetic and environmental influence on the covariance between verbal and nonverbal abilities: Twin and GCTA estimates

Table 2 shows genetic and environmental correlations between verbal and nonverbal abilities. The twin study estimates of genetic correlations are 0.71 at age 7 and 0.60 at age 12. As indicated by their standard errors, these genetic correlations are significant, and their magnitude is consistent with the literature in suggesting substantial genetic overlap between verbal and nonverbal abilities. At age 12, the GCTA estimate of the genetic correlation is 1.0, highly significant, and not significantly different from the twin study estimate of 0.60. However, at age 7, the GCTA estimate of the genetic correlation is 0.31, which does not reach significance with its large standard error of 0.32. Given the large standard errors of the GCTA estimates of genetic correlations, it is noteworthy that averaging the genetic correlations at the two ages yields the same genetic correlation of 0.66 for the twin and GCTA estimates.

As noted earlier, the phenotypic correlations between the verbal and nonverbal composites are 0.35 at age 7 and 0.45 at age 12. Genes are responsible for about half of the phenotypic correlation in the twin analyses at both ages 7 (51%) and 12 (51%). The same was true for GCTA at age 12 (53%), however at age 7 genes explained only 31% of the phenotypic correlation.

## 4 Discussion

Although the phenotypic correlation between verbal and nonverbal cognitive abilities is only 0.45 at age 12, our GCTA yielded a genetic correlation of 1.0 between verbal and nonverbal cognitive abilities. The current twin analysis, using the same sample and same measures as the GCTA, yielded a genetic correlation of 0.60 between verbal and nonverbal cognitive abilities at age 12. Despite the uncertainty of GCTA estimation, together, the GCTA and twin results at age 12 suggest that most of the genes that affect verbal ability also affect nonverbal ability and vice versa. Because verbal and nonverbal abilities represent a major bifurcation of general cognitive ability in the hierarchical model of intelligence, the high genetic correlation between verbal and nonverbal abilities implies that intelligence indexes generalist genes for cognitive abilities, that is, the same genes largely affect these disparate abilities. However, given that the twin-estimated genetic correlation is less than 1.0 leaves possibility that some genetic effects are not general across diverse cognitive abilities. As mentioned earlier, it is not surprising to find that some genes are specific to certain abilities — what is surprising is the great extent to which the same genes affect such different cognitive processes as those tapped by verbal tests of vocabulary and information and nonverbal tests of picture completion and progressive matrices.

At age 7, the results are not so clear. Although the twin study estimate of the genetic correlation between verbal and nonverbal abilities is just as high (0.71) as at age 12 (0.60), the GCTA estimate is only 0.31 at age 7, in contrast to 1.0 at age 12. It is possible that there is a developmental trend of increasing genetic correlations during childhood, as suggested in a few twin studies (Plomin, DeFries et al., 2013). However, our twin analyses do not support this hypothesis: Our genetic correlations are 0.71 at age 7 and 0.60 at age 12. Another possibility is methodological: Our tests at age 7 were administered via telephone, whereas at age 12 web-based tests were used. Although it is possible that the less traditional telephone tests show less genetic covariance as information processing is limited to the auditory modality, the phenotypic correlation between our verbal and nonverbal composites was only slightly less at age 7 (0.35) than at age 12 (0.45). That said, our twin analyses do not support this possibility because genetic correlations are similar at ages 7 and 12. This somewhat surprising result needs replication in larger sample before any further interpretations can be made, and the large standard error of 0.32 for the GCTA estimates of genetic correlation indicates the need for even larger samples for definitive results.

It should be mentioned that a genetic correlation between verbal and nonverbal abilities is pleiotropic at the descriptive level of suggesting that the genetic effects on verbal ability are correlated with the genetic effects on nonverbal abilities. As with any correlation, a genetic correlation does not specify the direction of causality — whether genes associated with verbal cause the genetic effects on nonverbal or vice versa, or whether genes associated with another trait cause the genetic effects on both verbal and nonverbal abilities.

One limitation of the present research is that it focuses on verbal and nonverbal composites rather than individual tests. However, because verbal and nonverbal cognitive abilities are a major bifurcation of general cognitive ability, they represent a key point to test the general gene hypothesis. In addition, the two verbal and two nonverbal tests at each age were designed to create a reliable measure of general cognitive ability at each age; we are stretching the data to create verbal and nonverbal composites, but the individual tests were not meant to be sufficiently reliable to stand on their own. Nonetheless, in the Supplementary data, we have included univariate and bivariate twin and GCTA results for the individual tests at both ages. Although the results for the individual tests bounce around considerably, especially for GCTA, they support the conclusions reached from the verbal and nonverbal composites presented in this report.

Another limitation of the present research is that it focuses on traditional behavioral tests, rather than tests of elementary processes such as information processing (Luo, Thompson, & Detterman, 2006), cognitive experimental tasks assessing constructs such as working memory (Baddeley, 2012), or measures of brain structure and function (Giedd & Rapoport, 2010). However, the value of our focus, at least as a starting point for research on generalist genes, is that these are the sorts of tests widely used to assess intelligence. Multivariate genetic research at the other levels is needed, as well as research farther upstream at all the ‘-omic’ levels between genes and brain, from transcriptomics and epigenomics to metabolomics and proteomics. Twin and GCTA designs are crucibles for testing the generalist gene hypothesis, and we predict that such research will find that genetic effects are general at all these steps between genes, brain and behavior. However, research at these other levels will be difficult because of the daunting demands for sample size, especially for GCTA analysis. Despite our sample size of about 2000 individuals genotyped on a million DNA markers, the standard errors of our GCTA estimates are large, especially for estimates of genetic correlations; sample sizes several times larger are needed.

One limitation of GCTA is that it only detects additive genetic effects of DNA variants, whereas the twin method captures nonadditive as well as additive genetic effects because identical twins are identical for DNA sequence variants. Details about additive and nonadditive genetic variance can be found elsewhere (e.g., Plomin, DeFries et al., 2013). Another limitation is that GCTA can only detect the effects of DNA variants tagged by (in linkage disequilibrium with) common SNPs (minor allele frequencies greater than 1%) incorporated in most DNA arrays. This limitation means that the effects of rare DNA variants are likely to be missed, unlike the twin method which summarizes the effects of all genetic variants. However, these limitations of GCTA – that it can only detect additive effects of common variants – imply that the comparison between the results of GCTA and twin studies provides important clues about how to identify genes responsible for the heritability of complex traits. To the extent that GCTA estimates of heritability are similar to twin study estimates of heritability, it follows that heritability is due to additive effects of common variants. This in turn indicates the extent to which genome-wide association studies, which have the same limitation as GCTA on additive effects of common SNPs, should be able to close the gap between genetic effects identified in genome-wide association studies and twin study heritability estimates, called the *missing heritability problem* (Maher, 2008). Our results at age 12 yield average GCTA estimates of genetic influence that are half the twin study heritability estimates, as reported previously for this sample (Plomin, Haworth et al., 2013). This finding suggests that additive effects of common variants can account for about half of the heritability of cognitive abilities but nonadditive effects and rare variants are also likely to be important.

GCTA is an important new tool to assess the net effect of genes on variance and covariance in cognitive abilities, but what is needed ultimately are the G, C, T, and A differences that are responsible for the strong genetic contribution to cognitive abilities. That is, nothing would advance the field more than identifying some of the many genes responsible for the heritability of intelligence. However, the identification of genes associated with cognitive abilities and all complex traits remains elusive (Chabris et al., 2012; Plomin, 2012). For example, the first genome-wide association studies of g (Davies et al., 2011; Davis et al., 2010) were powered to detect SNP associations with g that account for as little as 1% of the variance, but they came up empty-handed because the associations of largest effect account for less than 0.5% of the variance. Multivariate genetic research on cognitive abilities, including the present twin and GCTA results at age 12, suggests that most of the genetic action is general across diverse cognitive abilities rather than specific to a single ability. Intelligence is a good target for gene hunting because it indexes these generalist genes.

## Figures and Tables

**Table 1:** Twin and GCTA parameter estimates for verbal and nonverbal abilities at ages 7 and 12.

	Twin			GCTA	
	A	C	E	A (SE)	E (SE)
Age 7					
Verbal	.29 (.06)	.34 (.05)	.36 (.02)	.47 (.18)	.52 (.17)
Nonverbal	.21 (.07)	.28 (.05)	.50 (.03)	.26 (.17)	.74 (.17)

Age 12					
Verbal	.36 (.06)	.21 (.05)	.43 (.02)	.23 (.13)	.76 (.13)
Nonverbal	.42 (.06)	.16 (.05)	.42 (.02)	.15 (.14)	.84 (.14)

Standard error (SE) is shown in parentheses. Twin analyses were restricted to twin pairs for whom one member of the twin pair was included in GCTA. The twin analyses at age 7 were based on 734 MZ and 1146 DZ twin pairs for verbal and 742 MZ and 1164 DZ twin pairs for nonverbal; twin analyses at age 12 were based on 920 MZ and 1432 DZ twin pairs for verbal and 894 MZ and 1402 DZ twin pairs for nonverbal. The numbers of unrelated individuals in GCTA analyses were 1900 for verbal and 1917 for nonverbal at age 7 and 2496 for verbal and 2428 for nonverbal at age 12.

**Table 2:** Twin and GCTA genetic and environmental correlations between verbal and nonverbal abilities at ages 7 and 12.

	Twin			GCTA	
	r_A_	r_C_	r_E_	r_A_	r_E_
Age 7	.71 (.15)	.39 (.11)	.11 (.04)	.31 (.32)	.42 (.16)
Age 12	.60 (.09)	.79 (.13)	.17 (.03)	1.00 (.32)	.30 (.11)

r_A_ = additive genetic correlation; r_C_ = common or shared environmental correlation; r_E_ = residual or nonshared environmental correlation. Standard error (SE) is shown in parentheses. Sample sizes for twin analyses were 732 MZ pairs and 1141 DZ pairs at age 7 and 891 MZ pairs and 1390 DZ pairs at age 12. Sample sizes of unrelated individuals for GCTA analyses were 2496 at age 7 and 2428 at age 12. Genetic correlations were constrained not to exceed 1.0.
