# Supplementary material for: Intelligence indexes generalist genes for cognitive abilities
Source: Intelligence. 2013 Sep;41(5):560–5. doi: 10.1016/j.intell.2013.07.011 (PMC3928847; doi:10.1016/j.intell.2013.07.011)
Supplement: Supplementary file 1 — Supplementary material. [file mmc1.docx]

**Supplementary Online Materials**

**Supplementary Table 1.** Full results from univariate twin analyses.

|  | Composite | A | |  | C | |  | E | |  | SAMPLE SIZE | | |
| --- | --- | --- | --- | --- | --- | --- | --- | --- | --- | --- | --- | --- | --- |
| *trait* |  | *Estimate* | *SE* |  | *Estimate* | *SE* |  | *Estimate* | *SE* |  | *n.mz* | *n.dz* | *n.all* |
| *Age 7* |  |  |  |  |  |  |  |  |  |  |  |  |  |
| *Conceptual grouping* | Verbal | 0.15 | 0.08 |  | 0.18 | 0.06 |  | 0.67 | 0.03 |  | 741 | 1166 | 1911 |
| *Similarities* | Verbal | 0.17 | 0.07 |  | 0.29 | 0.06 |  | 0.54 | 0.03 |  | 736 | 1147 | 1887 |
| *Vocabulary* | Non-verbal | 0.23 | 0.06 |  | 0.36 | 0.05 |  | 0.41 | 0.02 |  | 728 | 1141 | 1873 |
| *Picture completion* | Non-verbal | 0.25 | 0.07 |  | 0.26 | 0.05 |  | 0.49 | 0.03 |  | 740 | 1155 | 1899 |
| *Verbal composite* | – | 0.29 | 0.06 |  | 0.34 | 0.05 |  | 0.36 | 0.02 |  | 734 | 1146 | 1884 |
| *Non-Verbal composite* | – | 0.21 | 0.07 |  | 0.28 | 0.05 |  | 0.50 | 0.03 |  | 742 | 1164 | 1910 |
|  |  |  |  |  |  |  |  |  |  |  |  |  |  |
| *Age 12* |  |  |  |  |  |  |  |  |  |  |  |  |  |
| *General knowledge* | Verbal | 0.26 | 0.06 |  | 0.29 | 0.04 |  | 0.45 | 0.02 |  | 921 | 1434 | 2359 |
| *Vocabulary* | Verbal | 0.29 | 0.07 |  | 0.15 | 0.05 |  | 0.56 | 0.03 |  | 882 | 1370 | 2256 |
| *Picture completion* | Non-verbal | 0.27 | 0.07 |  | 0.19 | 0.05 |  | 0.55 | 0.03 |  | 857 | 1324 | 2185 |
| *Ravens* | Non-verbal | 0.30 | 0.07 |  | 0.17 | 0.05 |  | 0.53 | 0.02 |  | 899 | 1411 | 2314 |
| *Verbal composite* | – | 0.36 | 0.06 |  | 0.21 | 0.05 |  | 0.43 | 0.02 |  | 920 | 1432 | 2356 |
| *Non-Verbal composite* | – | 0.42 | 0.06 |  | 0.16 | 0.05 |  | 0.42 | 0.02 |  | 894 | 1402 | 2300 |

Annotation: (A) – additive genetic influence; (C) – shared/common environmental influence; (E) – unique/non-shared environmental influence; (SE) – standard error; (n.mz) – number of Monozygotic twin pairs; (n.dz) – number of Dizygotic twin pairs; (n.all) – total number of twin pairs;

**Supplementary Table 2a.** A, C and E results from the bivariate twin analyses

|  |  | A_tr1 | |  | A_tr2 | |  | C_tr1 | |  | C_tr2 | |  | E_tr1 | |  | E_tr2 | |  | SAMPLE SIZE | | |
| --- | --- | --- | --- | --- | --- | --- | --- | --- | --- | --- | --- | --- | --- | --- | --- | --- | --- | --- | --- | --- | --- | --- |
| *tr_1* | *tr_2* | *Estimate* | *SE* |  | *Estimate* | *SE* |  | *Estimate* | *SE* |  | *Estimate* | *SE* |  | *Estimate* | *SE* |  | *Estimate* | *SE* |  | *n.mz* | *n.dz* | *n.all* |
| *Age 7* |  |  |  |  |  |  |  |  |  |  |  |  |  |  |  |  |  |  |  |  |  |  |
| *Conceptual grouping* | *Similarities* | 0.14 | 0.07 |  | 0.16 | 0.07 |  | 0.19 | 0.06 |  | 0.30 | 0.05 |  | 0.67 | 0.03 |  | 0.54 | 0.03 |  | 732 | 1145 | 1881 |
| *Conceptual grouping* | *Vocabulary* | 0.16 | 0.07 |  | 0.23 | 0.06 |  | 0.18 | 0.06 |  | 0.36 | 0.05 |  | 0.67 | 0.03 |  | 0.41 | 0.02 |  | 726 | 1139 | 1869 |
| *Conceptual grouping* | *Picture completion* | 0.15 | 0.08 |  | 0.26 | 0.07 |  | 0.18 | 0.06 |  | 0.26 | 0.05 |  | 0.67 | 0.03 |  | 0.49 | 0.03 |  | 737 | 1152 | 1893 |
| *Similarities* | *Vocabulary* | 0.19 | 0.06 |  | 0.23 | 0.05 |  | 0.28 | 0.05 |  | 0.37 | 0.04 |  | 0.53 | 0.02 |  | 0.40 | 0.02 |  | 720 | 1123 | 1847 |
| *Similarities* | *Picture completion* | 0.18 | 0.07 |  | 0.26 | 0.07 |  | 0.29 | 0.06 |  | 0.26 | 0.05 |  | 0.54 | 0.03 |  | 0.48 | 0.03 |  | 731 | 1134 | 1869 |
| *Vocabulary* | *Picture completion* | 0.23 | 0.06 |  | 0.26 | 0.07 |  | 0.37 | 0.05 |  | 0.26 | 0.05 |  | 0.40 | 0.02 |  | 0.48 | 0.03 |  | 725 | 1129 | 1858 |
| *Verbal* | *Non-verbal* | 0.29 | 0.05 |  | 0.23 | 0.07 |  | 0.35 | 0.05 |  | 0.28 | 0.05 |  | 0.36 | 0.02 |  | 0.50 | 0.03 |  | 732 | 1141 | 1877 |
|  |  |  |  |  |  |  |  |  |  |  |  |  |  |  |  |  |  |  |  |  |  |  |
| *Age 12* |  |  |  |  |  |  |  |  |  |  |  |  |  |  |  |  |  |  |  |  |  |  |
| *General knowledge* | *Vocabulary* | 0.27 | 0.05 |  | 0.30 | 0.06 |  | 0.28 | 0.04 |  | 0.15 | 0.05 |  | 0.45 | 0.02 |  | 0.56 | 0.02 |  | 881 | 1366 | 2251 |
| *General knowledge* | *Picture completion* | 0.27 | 0.06 |  | 0.28 | 0.07 |  | 0.29 | 0.04 |  | 0.18 | 0.05 |  | 0.45 | 0.02 |  | 0.54 | 0.02 |  | 857 | 1319 | 2180 |
| *General knowledge* | *Ravens* | 0.27 | 0.06 |  | 0.31 | 0.06 |  | 0.28 | 0.04 |  | 0.16 | 0.05 |  | 0.45 | 0.02 |  | 0.53 | 0.02 |  | 897 | 1400 | 2301 |
| *Vocabulary* | *Picture completion* | 0.29 | 0.07 |  | 0.28 | 0.07 |  | 0.15 | 0.05 |  | 0.18 | 0.05 |  | 0.56 | 0.03 |  | 0.54 | 0.02 |  | 853 | 1316 | 2173 |
| *Vocabulary* | *Ravens* | 0.29 | 0.07 |  | 0.31 | 0.06 |  | 0.15 | 0.05 |  | 0.16 | 0.05 |  | 0.56 | 0.03 |  | 0.53 | 0.02 |  | 878 | 1367 | 2249 |
| *Picture completion* | *Ravens* | 0.29 | 0.07 |  | 0.32 | 0.06 |  | 0.17 | 0.05 |  | 0.16 | 0.05 |  | 0.54 | 0.02 |  | 0.52 | 0.02 |  | 853 | 1321 | 2178 |
| *Verbal* | *Non-verbal* | 0.36 | 0.06 |  | 0.42 | 0.06 |  | 0.21 | 0.05 |  | 0.16 | 0.05 |  | 0.43 | 0.02 |  | 0.41 | 0.02 |  | 891 | 1390 | 2285 |

Annotation: (A_tr1/tr2) – additive genetic influence for trait 1 and 2 respectively; (C_tr1/tr2) – shared/common environmental influence for trait 1 and 2 respectively; (E_tr1/tr2) – unique/non-shared environmental influence for trait 1 and 2 respectively; (SE) – standard error; (n.mz) – number of Monozygotic twin pairs; (n.dz) – number of Dizygotic twin pairs; (n.all) – total number of twin pairs;

**Supplementary Table 2b.** A, C and E correlations from the bivariate twin models

|  |  | rG | |  | rC | |  | rE | |  | rPh | |  | C(G)* | |  | C(C) | |  | C(E) | |
| --- | --- | --- | --- | --- | --- | --- | --- | --- | --- | --- | --- | --- | --- | --- | --- | --- | --- | --- | --- | --- | --- |
| *tr_1* | *tr_2* | *Estimate* | *SE* |  | *Estimate* | *SE* |  | *Estimate* | *SE* |  | *Estimate* | *SE* |  | *Estimate* | *SE* |  | *Estimate* | *SE* |  | *Estimate* | *SE* |
| *Conceptual grouping* | *Similarities age 7* | 0.75 | 0.25 |  | 0.34 | 0.18 |  | 0.06 | 0.03 |  | 0.23 | 0.02 |  | 0.11 | 0.05 |  | 0.08 | 0.04 |  | 0.03 | 0.02 |
| *Conceptual grouping* | *Vocabulary age 7* | 0.76 | 0.19 |  | 0.22 | 0.17 |  | 0.00 | 0.04 |  | 0.20 | 0.02 |  | 0.14 | 0.05 |  | 0.05 | 0.04 |  | 0.00 | 0.02 |
| *Conceptual grouping* | *Picture completion age 7* | 0.27 | 0.34 |  | 0.31 | 0.21 |  | 0.03 | 0.04 |  | 0.14 | 0.02 |  | 0.05 | 0.05 |  | 0.07 | 0.04 |  | 0.02 | 0.02 |
| *Similarities* | *Vocabulary age 7* | 1.00 | 0.07 |  | 0.69 | 0.07 |  | 0.18 | 0.03 |  | 0.51 | 0.01 |  | 0.21 | 0.04 |  | 0.22 | 0.04 |  | 0.08 | 0.02 |
| *Similarities* | *Picture completion age 7* | 0.32 | 0.26 |  | 0.37 | 0.15 |  | 0.08 | 0.03 |  | 0.21 | 0.02 |  | 0.07 | 0.05 |  | 0.10 | 0.04 |  | 0.04 | 0.02 |
| *Vocabulary* | *Picture completion age 7* | 0.54 | 0.19 |  | 0.32 | 0.12 |  | 0.11 | 0.04 |  | 0.28 | 0.02 |  | 0.13 | 0.05 |  | 0.10 | 0.04 |  | 0.05 | 0.02 |
| *General knowledge* | *Vocabulary age 12* | 1.00 | 0.07 |  | 0.61 | 0.14 |  | 0.06 | 0.03 |  | 0.44 | 0.01 |  | 0.28 | 0.04 |  | 0.12 | 0.03 |  | 0.03 | 0.01 |
| *General knowledge* | *Picture completion age 12* | 0.59 | 0.18 |  | 0.31 | 0.16 |  | 0.05 | 0.03 |  | 0.26 | 0.01 |  | 0.16 | 0.04 |  | 0.07 | 0.04 |  | 0.02 | 0.02 |
| *General knowledge* | *Ravens age 12* | 0.37 | 0.14 |  | 0.77 | 0.13 |  | 0.18 | 0.03 |  | 0.36 | 0.01 |  | 0.11 | 0.04 |  | 0.16 | 0.04 |  | 0.09 | 0.02 |
| *Vocabulary* | *Picture completion age 12* | 0.51 | 0.18 |  | 0.51 | 0.24 |  | 0.08 | 0.03 |  | 0.27 | 0.01 |  | 0.14 | 0.05 |  | 0.08 | 0.04 |  | 0.04 | 0.02 |
| *Vocabulary* | *Ravens age 12* | 0.76 | 0.13 |  | 0.47 | 0.24 |  | 0.11 | 0.03 |  | 0.36 | 0.01 |  | 0.23 | 0.05 |  | 0.07 | 0.04 |  | 0.06 | 0.02 |
| *Picture completion* | *Ravens age 12* | 0.86 | 0.11 |  | 0.32 | 0.24 |  | 0.05 | 0.03 |  | 0.34 | 0.01 |  | 0.26 | 0.05 |  | 0.05 | 0.04 |  | 0.02 | 0.02 |
| *Verbal* | *Non-verbal age 7* | 0.71 | 0.15 |  | 0.39 | 0.11 |  | 0.11 | 0.04 |  | 0.35 | 0.02 |  | 0.18 | 0.04 |  | 0.12 | 0.04 |  | 0.04 | 0.02 |
| *Verbal* | *Non-verbal age 12* | 0.60 | 0.09 |  | 0.79 | 0.13 |  | 0.17 | 0.03 |  | 0.45 | 0.01 |  | 0.23 | 0.04 |  | 0.14 | 0.04 |  | 0.07 | 0.01 |

Annotation: (rG) – genetic correlation; (rC) – common environment correlation; (rPh) – phenotypic correlation; (C(G)) – proportion of the phenotypic correlation due to genetic covariation; (C(C)) – proportion of the phenotypic correlation explained by covariation of common environment; (C(E)) – proportion of the phenotypic correlation explained by unique environment;

* This estimate is directly comparable to GCTA’s ‘C(G)_tr12’ estimate;

**Supplementary Table 3.** The genetic and residual results from the univariate GCTA analyses for all the tests.

| Variable | Composite | V(G) | SE | V(e) | SE | Vp | SE | V(G)/Vp | SE | logL | logL0 | LRT | df | Pval | n |
| --- | --- | --- | --- | --- | --- | --- | --- | --- | --- | --- | --- | --- | --- | --- | --- |
| *Age 7* |  |  |  |  |  |  |  |  |  |  |  |  |  |  |  |
| *Vocabulary* | *Verbal* | 0.54 | 0.18 | 0.45 | 0.18 | 1.00 | 0.03 | 0.55 | 0.18 | -935.50 | -940.15 | 9.29 | 1 | 0.00 | 1893 |
| *Conceptual grouping* | *Verbal* | 0.22 | 0.17 | 0.77 | 0.17 | 0.99 | 0.03 | 0.23 | 0.17 | -945.88 | -946.84 | 1.94 | 1 | 0.08 | 1919 |
| *Similarities* | *Non-verbal* | 0.29 | 0.18 | 0.70 | 0.18 | 0.99 | 0.03 | 0.29 | 0.18 | -940.87 | -942.18 | 2.63 | 1 | 0.05 | 1901 |
| *Picture completion* | *Non-verbal* | 0.18 | 0.17 | 0.82 | 0.17 | 1.00 | 0.03 | 0.18 | 0.17 | -947.10 | -947.67 | 1.13 | 1 | 0.10 | 1909 |
| *Verbal composite* | *–* | 0.47 | 0.18 | 0.52 | 0.17 | 0.99 | 0.03 | 0.47 | 0.18 | -938.78 | -942.39 | 7.21 | 1 | 0.00 | 1900 |
| *Non-Verbal composite* | *–* | 0.26 | 0.17 | 0.74 | 0.17 | 0.99 | 0.03 | 0.26 | 0.17 | -949.70 | -950.93 | 2.46 | 1 | 0.06 | 1917 |
|  |  |  |  |  |  |  |  |  |  |  |  |  |  |  |  |
| *Age 12* |  |  |  |  |  |  |  |  |  |  |  |  |  |  |  |
| *General knowledge* | *Verbal* | 0.35 | 0.13 | 0.64 | 0.13 | 0.99 | 0.03 | 0.35 | 0.13 | -1232.25 | -1235.83 | 7.17 | 1 | 0.00 | 2499 |
| *Vocabulary* | *Verbal* | 0.07 | 0.14 | 0.91 | 0.14 | 0.99 | 0.03 | 0.07 | 0.14 | -1171.92 | -1172.07 | 0.30 | 1 | 0.30 | 2377 |
| *Picture completion* | *Non-verbal* | 0.09 | 0.14 | 0.90 | 0.14 | 0.99 | 0.03 | 0.09 | 0.14 | -1150.01 | -1150.22 | 0.42 | 1 | 0.30 | 2317 |
| *Ravens* | *Non-verbal* | 0.02 | 0.13 | 0.98 | 0.13 | 0.99 | 0.03 | 0.02 | 0.13 | -1208.26 | -1208.27 | 0.02 | 1 | 0.40 | 2434 |
| *Verbal composite* | *–* | 0.23 | 0.13 | 0.76 | 0.13 | 0.99 | 0.03 | 0.23 | 0.13 | -1230.10 | -1231.71 | 3.22 | 1 | 0.04 | 2496 |
| *Non-Verbal composite* | *–* | 0.15 | 0.14 | 0.84 | 0.14 | 0.99 | 0.03 | 0.15 | 0.14 | -1204.32 | -1204.97 | 1.29 | 1 | 0.10 | 2428 |

Annotation: (V(G)) – variance due to additive genetic influences; (V(e)) – residual variance; (Vp) – phenotypic variance; (V(G)/Vp) – proportion of the phenotypic variance due to additive genetic influences; (logL) – log likelihood of the alternative model; (logL0) – log likelihood of the null model; (LRT) – likelihood ratio; (df) – degrees of freedom; (Pval) – p-value; (n) – sample size;

**Supplementary Table 4.** Full results from the bivariate GCTA analyses for all scales

|  |  | V(G)_tr1 | SE | V(G)_tr2 | SE | C(G)_tr12 | SE | V(e)_tr1 | SE | V(e)_tr2 | SE | C(e)_tr12 | SE | Vp_tr1 | SE | Vp_tr2 | SE | V(G)/Vp_tr1 | SE | V(G)/Vp_tr2 | SE | rG | SE | rE | SE | logL | n/pairs |
| --- | --- | --- | --- | --- | --- | --- | --- | --- | --- | --- | --- | --- | --- | --- | --- | --- | --- | --- | --- | --- | --- | --- | --- | --- | --- | --- | --- |
| *Age 7* | |  |  |  |  |  |  |  |  |  |  |  |  |  |  |  |  |  |  |  |  |  |  |  |  |  |  |
| *Conceptual grouping* | *Similarities* | 0.23 | 0.17 | 0.30 | 0.18 | 0.14 | 0.13 | 0.76 | 0.17 | 0.70 | 0.18 | 0.10 | 0.13 | 0.99 | 0.03 | 1.00 | 0.03 | 0.23 | 0.17 | 0.30 | 0.18 | 0.55 | 0.43 | 0.14 | 0.16 | -1828.54 | 1892 |
| *Conceptual grouping* | *Vocabulary* | 0.23 | 0.17 | 0.52 | 0.18 | 0.14 | 0.13 | 0.76 | 0.17 | 0.48 | 0.18 | 0.07 | 0.12 | 0.99 | 0.03 | 1.00 | 0.03 | 0.23 | 0.17 | 0.52 | 0.18 | 0.42 | 0.34 | 0.11 | 0.20 | -1838.01 | 1907 |
| *Conceptual grouping* | *Picture completion* | 0.22 | 0.17 | 0.18 | 0.17 | 0.09 | 0.12 | 0.77 | 0.17 | 0.82 | 0.17 | 0.06 | 0.12 | 0.99 | 0.03 | 1.00 | 0.03 | 0.22 | 0.17 | 0.18 | 0.17 | 0.46 | 0.60 | 0.07 | 0.15 | -1869.73 | 1900 |
| *Similarities* | *Vocabulary* | 0.30 | 0.18 | 0.50 | 0.18 | 0.27 | 0.14 | 0.70 | 0.18 | 0.50 | 0.18 | 0.25 | 0.14 | 1.00 | 0.03 | 1.00 | 0.03 | 0.30 | 0.18 | 0.50 | 0.18 | 0.70 | 0.22 | 0.42 | 0.17 | -1590.32 | 1884 |
| *Similarities* | *Picture completion* | 0.29 | 0.18 | 0.17 | 0.17 | -0.02 | 0.13 | 0.71 | 0.18 | 0.83 | 0.17 | 0.24 | 0.13 | 0.99 | 0.03 | 1.00 | 0.03 | 0.29 | 0.18 | 0.17 | 0.17 | -0.08 | 0.59 | 0.31 | 0.15 | -1838.79 | 1890 |
| *Vocabulary* | *Picture completion* | 0.52 | 0.18 | 0.18 | 0.17 | 0.12 | 0.13 | 0.48 | 0.18 | 0.81 | 0.17 | 0.18 | 0.13 | 1.00 | 0.03 | 1.00 | 0.03 | 0.52 | 0.18 | 0.18 | 0.17 | 0.40 | 0.36 | 0.28 | 0.18 | -1792.87 | 2380 |
| *Verbal* | *Non-verbal* | 0.46 | 0.18 | 0.26 | 0.17 | 0.11 | 0.13 | 0.54 | 0.17 | 0.74 | 0.17 | 0.27 | 0.13 | 1.00 | 0.03 | 0.99 | 0.03 | 0.46 | 0.18 | 0.26 | 0.17 | 0.31 | 0.32 | 0.42 | 0.16 | -1745.18 | 1878 |
|  |  |  |  |  |  |  |  |  |  |  |  |  |  |  |  |  |  |  |  |  |  |  |  |  |  |  |  |
| *Age 12* | |  |  |  |  |  |  |  |  |  |  |  |  |  |  |  |  |  |  |  |  |  |  |  |  |  |  |
| *General knowledge* | *Vocabulary* | 0.35 | 0.13 | 0.08 | 0.13 | 0.11 | 0.10 | 0.64 | 0.13 | 0.91 | 0.14 | 0.32 | 0.10 | 0.99 | 0.03 | 0.99 | 0.03 | 0.35 | 0.13 | 0.08 | 0.14 | 0.67 | 0.54 | 0.42 | 0.10 | -2156.30 | 2377 |
| *General knowledge* | *Picture completion* | 0.35 | 0.13 | 0.11 | 0.14 | 0.21 | 0.10 | 0.64 | 0.13 | 0.89 | 0.14 | 0.08 | 0.10 | 0.99 | 0.03 | 0.99 | 0.03 | 0.35 | 0.13 | 0.11 | 0.14 | 1.00 | 0.70 | 0.10 | 0.13 | -2281.57 | 2433 |
| *General knowledge* | *Ravens* | 0.35 | 0.13 | 0.03 | 0.13 | 0.21 | 0.10 | 0.64 | 0.13 | 0.96 | 0.13 | 0.17 | 0.10 | 0.99 | 0.03 | 0.99 | 0.03 | 0.35 | 0.13 | 0.04 | 0.13 | 1.00 | 3.16 | 0.21 | 0.11 | -2256.78 | 2316 |
| *Vocabulary* | *Picture completion* | 0.07 | 0.14 | 0.11 | 0.14 | 0.02 | 0.10 | 0.91 | 0.14 | 0.88 | 0.14 | 0.27 | 0.10 | 0.99 | 0.03 | 0.99 | 0.03 | 0.07 | 0.14 | 0.11 | 0.14 | 0.27 | 1.01 | 0.29 | 0.10 | -2219.69 | 1896 |
| *Vocabulary* | *Ravens* | 0.06 | 0.13 | 0.03 | 0.13 | 0.10 | 0.10 | 0.92 | 0.14 | 0.97 | 0.13 | 0.26 | 0.10 | 0.99 | 0.03 | 0.99 | 0.03 | 0.06 | 0.14 | 0.03 | 0.13 | 0.00 | 0.00 | 0.27 | 0.09 | -2214.08 | 2424 |
| *Picture completion* | *Ravens* | 0.10 | 0.14 | 0.02 | 0.13 | 0.08 | 0.10 | 0.90 | 0.14 | 0.97 | 0.13 | 0.26 | 0.10 | 1.00 | 0.03 | 0.99 | 0.03 | 0.10 | 0.14 | 0.02 | 0.13 | 1.00 | 5.41 | 0.28 | 0.10 | -2218.63 | 2317 |
| *Verbal* | *Non-verbal* | 0.23 | 0.13 | 0.22 | 0.14 | 0.24 | 0.10 | 0.76 | 0.13 | 0.78 | 0.14 | 0.23 | 0.10 | 0.99 | 0.03 | 1.00 | 0.03 | 0.23 | 0.13 | 0.22 | 0.14 | 1.00 | 0.32 | 0.30 | 0.11 | -2146.60 | 2320 |

Annotation: V(G) – variance explained by genetic factors for trait 1 and trait 2 (tr1, tr2); C(G) – covariance between trait 1 and 2 explained by genetic factors; V(e) – residual variance for trait 1 and trait 2; C(e) – residual covariance between trait 1 and trait 2; Vp – phenotypic variance for trait 1 and trait 2; V(G) / Vp – proportion of the phenotypic variance explained by genetic factors for trait 1 and trait 2; r_G_ – genetic correlation between trait 1 and trait 2 (constrained not to exceed 1.0); logL – log likelihood estimation of the model; n – number of individuals with data for both trait 1 and trait 2; values in parentheses are standard errors.

*GCTA incorporates full-information maximum likelihood that uses the full sample of more than 2900 individuals with data on trait 1 or trait 2. However, the variance estimates for each trait are based on individuals with data for that trait and the covariance estimates are based on individuals with data for both traits. The n reported in the last column is the most conservative, i.e., the n that was used for the estimation of the covariance.

** The current version of GCTA does not report the environmental correlation or its standard error. The environmental correlation was derived here from the GCTA estimates using the following algorithm: C(e)_tr12 / ( √V(e)_tr1 * √V(e)_tr2), whereas the standard error was calculated using: Var(re) = re * re * (VarVe1/(4*Ve1*Ve1) + VarVe2/(4*Ve2*Ve2) + VarCe/(Ce*Ce) + CovVe1Ve2/(2*Ve1*Ve2) - CovVe1Ce/(Ve1*Ce) - CovVe2Ce/(Ve2*Ce)); SE(re) = sqrt[Var(re)], where re is the environmental correlation, Ve1 is the residual variance for trait 1, Ce is the residual covariance between two traits, VarVe1 is the sampling variance for Ve1 (residual variance for trait 1), VarCe is the sampling variance for Ce, CovVe1Ve2 is the sampling covariance between Ve1 and Ve2, and CovVe1Ce is the sampling covariance between Ve1 and Ce.
